# Extracellular Vesicles Secreted by TGF-*β*1-Treated Mesenchymal Stem Cells Promote Fracture Healing by SCD1-Regulated Transference of LRP5

**DOI:** 10.1155/2023/4980871

**Published:** 2023-03-15

**Authors:** Zihui Zhou, Chenyang Guo, Xulong Sun, Zhengwei Ren, Jie Tao

**Affiliations:** Department of Orthopedics, Shanghai General Hospital, Shanghai Jiaotong University School of Medicine, Shanghai Jiaotong University, Shanghai 200080, China

## Abstract

Bone fracture repair is a multiphased regenerative process requiring paracrine intervention throughout the healing process. Mesenchymal stem cells (MSCs) play a crucial role in cell-to-cell communication and the regeneration of tissue, but their transplantation is difficult to regulate. The paracrine processes that occur in MSC-derived extracellular vesicles (MSC-EVs) have been exploited for this study. The primary goal was to determine whether EVs secreted by TGF-*β*1-stimulated MSCs (MSC^TGF-*β*1^-EVs) exhibit greater effects on bone fracture healing than EVs secreted by PBS-treated MSCs (MSC^PBS^-EVs). Our research was conducted using an *in vivo* bone fracture model and *in vitro* experiments, which included assays to measure cell proliferation, migration, and angiogenesis, as well as *in vivo* and *in vitro* gain/loss of function studies. In this study, we were able to confirm that SCD1 expression and MSC-EVs can be induced by TGF-*β*1. After MSC^TGF-*β*1^-EVs are transplanted in mice, bone fracture repair is accelerated. MSC^TGF-*β*1^-EV administration stimulates human umbilical vein endothelial cell (HUVEC) angiogenesis, proliferation, and migration *in vitro*. Furthermore, we were able to demonstrate that SCD1 plays a functional role in the process of MSC^TGF-*β*1^-EV-mediated bone fracture healing and HUVEC angiogenesis, proliferation, and migration. Additionally, using a luciferase reporter assay and chromatin immunoprecipitation studies, we discovered that SREBP-1 targets the promoter of the SCD1 gene specifically. We also discovered that the EV-SCD1 protein could stimulate proliferation, angiogenesis, and migration in HUVECs through interactions with LRP5. Our findings provide evidence of a mechanism whereby MSC^TGF-*β*1^-EVs enhance bone fracture repair by regulating the expression of SCD1. The use of TGF-*β*1 preconditioning has the potential to maximize the therapeutic effects of MSC-EVs in the treatment of bone fractures.

## 1. Introduction

The repair of bone fractures includes bone remodeling, angiogenesis, and formation of cartilage callus [1, 2]. Both the regeneration of bone and the paracrine communication that take place during this process rely heavily on angiogenesis [3, 4]. It facilitates the process for immune cells and bone precursor cells to be transported to the damaged area, and it boosts the delivery of oxygen and nutrients to the callus that is healing [5, 6].

Mesenchymal stem cells (MSCs) are multipotent stromal cells that have the potential to differentiate into a wide range of mesenchymal tissues, such as bone and cartilage, and several other tissues [7, 8]. It is speculated that MSCs work in tandem with innate and adaptive immune systems, osteoblasts, and osteoclasts to repair bone injury and restore bone function following a bone fracture [9, 10]. At the site of injury, MSCs produce bioactive compounds and vesicles containing proteins, nucleic acids, and lipids [11, 12].

Cell-to-cell communication at the site of injury is thought to be mediated by extracellular vesicles (EVs) that migrate through cells [13, 14]. MSC-derived EVs (MSC-EVs) carry a cargo of RNA and proteins that stimulate multiple physiological processes, including angiogenesis and extracellular matrix remodeling, to boost the process of tissue repair [15]. Human MSC-EVs are thought to participate in tissue repair through the activation of the transforming growth factor- (TGF-) *β* pathway [16, 17]. In addition, TGF-*β*1 is recognized to have a crucial role in the process of angiogenesis in humans. Furthermore, defects in associated pathways can lead to vascular diseases such as hereditary hemorrhagic telangiectasia [18]. However, it is unclear whether TGF-*β*1-stimulated MSCs secreting EVs (MSC^TGF-*β*1^-EVs) can accelerate bone fracture healing as well as whether such an improvement is dependent by EV-mediated signaling.

There are indications that the sterol-responsive element-binding protein- (SREBP-) 1 transcription factor is involved in the regulation of the TGF-*β*1 signaling pathway [19]. Recent evidence suggests that SREBP-1 influences TGF-*β*1 signaling through the exosome regulation of TGF-*β*1 receptor-1 [20]. SREBP-1 is upregulated with the suppression of stearoyl-coenzyme A desaturase-1 (SCD1) expression [21], a protein associated with osteogenesis and fracture risk in menopausal women with diabetes [22, 23].

In a series of *in vitro* and *in vivo* gain and loss of function experiments, we confirmed that MSC^TGF-*β*1^-EV-derived SCD1 has a functional role in the bone fracture healing process. MSC^TGF-*β*1^-EVs promoted angiogenesis, proliferation, and migration, whereas the knockdown of SREBP-1 resulted in a significant decrease of SCD1 in MSCs and EVs, abolishing the effects of TGF-*β*1. In the treatment of bone fractures, the use of MSC^TGF-*β*1^-EVs may prove to be an effective and promising technique.

## 2. Methods

### 2.1. Cell Culture and TGF-*β*1 Treatment

According to the findings of a previous investigation [24], MSCs were isolated from the umbilical cord tissue of human donors (*n* = 3, ages 23–29 years) who were in good condition. Briefly, umbilical cords that were collected from healthy neonatal deliveries were washed and then cut into pieces that were 10 mm^3^ in volume after the cord vessels were removed. The pieces were cultured in Dulbecco's modified Eagle's medium (DMEM) containing 10% fetal bovine serum (FBS, Gibco) and 1% penicillin-streptomycin (Invitrogen, Carlsbad, CA, USA) antibiotics at 37°C with 5% CO_2_ until they reached 70–80% confluency. Flow cytometry (FACSCalibur, BD Biosciences, San Jose, California, USA) was utilized to validate the expression of MSC surface markers. The markers CD73, CD73, CD90, CD14, CD34, and CD45 were utilized for this validation (BD Biosciences, San Jose, CA, USA). In this study, MSCs were primed with 10 ng/mL TGF-*β*1 or treated with PBS as vehicle control for 24 h. Human umbilical vein endothelial cells (HUVECs) were obtained from the American Type Culture Collection (CRL-1730, Rockville, MD, USA) and cultured in DMEM with 10% FBS at 37°C with 5% CO_2_ and deprived of serum for 24 h before treatment with TGF-*β*1 (10 ng/mL). This study was approved by the Ethics Committee of Shanghai General Hospital and conducted in accordance with the Declaration of Helsinki.

### 2.2. EV Isolation and Identification

MSCs were allowed to reach 80% confluency and then transferred to EV-depleted FBS for 48 h. The media was collected by centrifugation at 2,000 × *g* for 10 min at 4°C, and then, cellular debris was removed by filtration using a 0.22 *μ*m sterile filter (Millipore, Burlington, MA, USA). The filtered supernatant was concentrated to 200 *μ*L in an Amicon Ultra-15 Centrifugal Filter Unit (Millipore, Burlington, MA, USA) at 4,000 × *g*. EVs were recovered by ultracentrifugation at 100,000 × *g* for 60 min at 4°C in an Optima L-100 XP Ultracentrifuge (Beckman Coulter, Indianapolis, IN, USA). The fraction containing the EVs was verified with the EV markers TSG101, CD63, CD81, and HSP70 and by using transmission electron microscopy (TEM; Tecnai 12, Philips, Best, The Netherlands).

### 2.3. Femoral Fracture Model and X-Ray Imaging

The Animal Research Committee at Shanghai Jiao Tong University Affiliated Sixth People's Hospital reviewed and gave its approval to all of the experiments that involved animals. The murine femoral fracture was carried out as described previously [25]. Briefly, Kirschner's wire (K-wire; 1.0 mm) was inserted into the femoral marrow cavity of anesthetized mice (C57BL/6 background, 10–12 weeks, *n* = 36) and bone forceps were used to create a mid-diaphyseal fracture. The mice were divided into three groups (*n* = 12 in each group): PBS, MSC^PBS^-EV, and MSC^TGF-*β*1^-EV groups. Next, MSC^PBS^-EVs or MSC^TGF-*β*1^-EVs (a total of 200 *μ*g of EV protein was precipitated in a volume of 200 *μ*L of PBS) or an equal volume of PBS was injected immediately near the fracture on days 3, 5, and 7 after surgery. The wounds were closed with sutures, and the mice received daily buprenorphine to control pain postsurgery. The progress of the fractures was monitored in the Faxitron MX-20 X-ray system (Faxitron, Tucson, AZ, USA) 21 d after surgery. The K-wire was removed, and femurs were collected from euthanized mice, fixed in 4% paraformaldehyde for 24 h, decalcified in 10% ethylenediaminetetraacetic acid (EDTA), and embedded in paraffin for further analysis.

### 2.4. Micro-Computed Tomography (CT) Imaging

Femurs fixed in 4% paraformaldehyde were scanned by micro-CT at a resolution of 18 *μ*m using a SkyScan 1172 (Bruker, Billerica, MA, USA) at 50 kV and 200 *μ*A. Three-dimensional images were constructed and bone morphometric parameters were obtained using a CT analyzer (Bruker, Billerica, MA, USA). Assessment of micro-CT scans of the samples from the PBS, MSC^PBS^-EV, and MSC^TGF-*β*1^-EV groups, sacrificed at 21 d postoperatively, was used to quantify the between-group differences in new bone formation at the osteotomy site. The following new bone structural parameters were calculated and statistically analyzed from the region of interest at the osteotomy site: bone volume density (BV/TV, %), trabecular number (Tb.N, mm^−1^), trabecular thickness (Tb.Th, mm), trabecular spacing (Tb.Sp, mm), and bone mineral density (BMD) [26].

### 2.5. Micro-CT Analysis of Angiogenesis at the Fracture Sites

Micro-CT examination using a contrast agent was utilized to investigate the vascular networks in the area of the fractures. In a nutshell, a radiopaque silicone rubber compound was perfused into the heart prior to the removal of the fractured femurs. After that, the micro-CT system (SkyScan 1172, Bruker) was used to scan the femurs after they had been removed. After being submerged in 10% EDTA solution for 10 d, they were examined once more to reveal the callus's vascular structure. The use of a CT analyzer (Bruker) allowed for the generation of three-dimensional reconstructions.

### 2.6. Three-Point Bending Mechanical Test

A mechanical test was performed within 24 h of the sacrifice at room temperature. To determine the biomechanical properties of the femur samples, they were subjected to three-point bending using a three-point bending device (H25KS, Hounsfield Test Equipment, Surrey, UK). The femur samples were loaded in the anterior-posterior direction at a rate of 5 mm per minute until failure was achieved. The Vernier graphical analysis software was used to examine the ultimate load, stiffness, and energy to failure of the material.

### 2.7. Histochemical Analysis

Hematoxylin and eosin (H&E), toluidine blue (TB), and safranin O-fast green staining were performed on sections of femur tissue deparaffinized with xylene. To stain with H&E, sections were stained in hematoxylin for 5 min and eosin for 2 min and then rinsed briefly in water. For TB staining, sections were incubated in TB for 2–3 min and then rinsed with xylene. For safranin O-fast green staining, sections were stained with 0.02% fast green for 1 min, 1.0% acetic acid for 30 s, and 1.0% safranin O for 10 min and rinsed in xylene.

### 2.8. Immunofluorescence Staining

Following the deparaffinization of the sections in xylene, they were placed at room temperature for an overnight incubation with primary antibodies against Ki-67 and CD31 (1 : 500, Abcam, Cambridge, UK). Goat secondary antibodies conjugated with Alexa Fluor 488 and Alexa Flour 594 (Jackson ImmunoResearch, West Grove, PA, USA) were added, and the samples were incubated for 1 h at room temperature. Nuclei were stained with DAPI, and fluorescent images were obtained using a fluorescence microscope. All experiments were performed in triplicate.

### 2.9. Real-Time PCR

Total RNA was extracted from cells and EVs using TRIzol reagent (Invitrogen, Carlsbad, CA, USA) following the manufacturer's instructions. A Reverse Transcription Kit (Toyobo, Osaka, Japan) was used to synthesize cDNA. Real-time PCR was conducted on an ABI 7900 Real-Time PCR system (Applied Biosystems, Foster City, CA, USA) using SYBR Green PCR master mix from Applied Biosystems (Foster City, CA, USA). Levels of expression were normalized to GAPDH and evaluated using the 2^−*ΔΔ*CT^ method. The primer sequences are listed as follows: SCD1-F: GAGGCACCTACATTGGATGCT, SCD1-R: CGTAGACATAGGACCGCTCA; SREBP-1-F: ACCATCGGCACCCGCTGCTTTAAAGAT, SREBP-1-R: TGAATGGTGGCTGCTGAGTGTTTCCTG; and GAPDH-F: CTCACCGGATGCACCAATGTT, GAPDH-R: CGCGTTGCTCACAATGTTCAT.

### 2.10. Cell Counting Kit-8 (CCK-8) Assay

HUVEC proliferation was assessed using CCK-8 (Sigma-Aldrich, St. Louis, MO, USA). The cells (2.0 × 10^3^ cells/100 *μ*L medium) were cocultured with either PBS or 100 *μ*g/mL of MSC^PBS^-EVs or MSC^TGF-*β*1^-EVs. After 24, 48, or 72 h, the cells in each well were incubated for 2 h at 37°C with CCK-8 (10 *μ*L/well) solution, and the optical density was read on a microplate reader at 450 nm. All experiments were performed in triplicate.

### 2.11. 5-Ethynyl-2-Deoxyuridine (EdU) Assay

Following the instructions from the manufacturer, an EdU assay kit (Thermo Fisher Scientific, Waltham, MA, USA) was used to measure proliferation of cells. The cells (2.0 × 10^4^) were either visualized with fluorescence microscopy (Carl Zeiss Microscopy GmbH, Jena, Germany) or counted by flow cytometry (FACSCalibur) with an iClick EdU Andy Fluor 647 Flow Cytometry Assay Kit (Genecopoeia, Germantown, MD, USA). All experiments were performed in triplicate.

### 2.12. Tube Formation Assay

The angiogenic properties of EV-treated HUVECs were determined by tube formation using Matrigel (BD Biosciences, San Jose, CA). EV-treated HUVECs (2.0 × 10^4^) were seeded onto Matrigel-coated 96-well plates. Tube formation was observed under an optical microscope 6 h after plating, and the lengths of tubes were measured randomly in five separate fields using ImageJ software (National Institutes of Health, Bethesda, MD, USA). All experiments were performed in triplicate.

### 2.13. Migration Assays

For the purpose of determining the migration of HUVECs, a Transwell assay was utilized. The cells (2.0 × 10^5^) were seeded into the upper chamber of a 24-well Transwell plate (Corning, NY, USA). The cells that migrated to the lower chamber were stained with crystal violet. A wound assay was also performed to determine the level of migration in EV-treated cells. A confluent layer of cells (2.0 × 10^5^ cells/well) was scratched with a pipette tip, and the level of migration was observed 12 h later. All experiments were performed in triplicate.

### 2.14. Luciferase Reporter Assay

To determine the possible interaction between SREBP-1 and SCD1 promoter, we used a luciferase reporter assay. The putative SREBP-1 binding site was mutated in the promoter of SCD1, and the mutated sequence and wild-type sequence were inserted separately into pmir-GLO-promoter vectors (Promega, Madison, WI, USA). SREBP-1 was inserted into a reporter vector, and the constructs were transfected into cells. A Dual-Luciferase Reporter Kit (Promega, Madison, WI, USA) was used to measure the luciferase activity in transfected cells and normalized to Renilla. All experiments were performed in triplicate.

### 2.15. Chromatin Immunoprecipitation (ChIP) Assay

To confirm the luciferase reporter assay results, we conducted a ChIP assay. The cells were fixed in 1% formaldehyde and then centrifuged at 800 × *g* for 5 min at 4°C. The cells were lysed, and chromatin was cut up into small pieces using an Ultrasonic Cell Disruptor (Covaris, Waltham, MA, USA). SREBP-1 binding to the SCD1 promoter was confirmed by adding anti-SREBP-1 antibodies (Abcam, Cambridge, MA, USA), and the target sequence in the immunoprecipitated fragments was detected by PCR.

### 2.16. Western Blot Analysis

Proteins were extracted from cells using RIPA buffer (89900, Thermo Fisher Scientific, Grand Island, NY, USA), and protein concentration was measured by using a BCA assay (23225, Thermo Fisher Scientific), and equal quantities of protein were separated by SDS-PAGE. Proteins were transferred to polyvinylidene fluoride (PVDF) membranes (Millipore) and blocked with 5% bovine serum albumin. Blocked PVD membranes were incubated overnight at 4°C with primary antibodies at dilutions recommended by the manufacturers. The following primary antibodies were used for western blot analysis: anti-SCD1 (Abcam, ab236868, 1 : 1,000), anti-GAPDH (Abcam, ab8245, 1: 1,000), anti-TSG101 (Abcam, ab83, 1 : 1,000), anti-CD81 (Abcam, ab286173, 1 : 500), anti-CD63 (Abcam, ab1318, 1 : 1,000), anti-HSP70 (Abcam, ab2787, 1 : 1,000), anti-*β*-actin (Abcam, ab7817, 1 : 2,000), anti-SREBP-1 (Abcam, ab28481, 1 : 1,000), anti-LRP5 (Abcam, ab223203, 1 : 1,000), and anti-LRP6 (Abcam, ab231779, 1 : 1,000). Membranes were then incubated with secondary antibodies for 1 h at room temperature and immunoreactive bands were detected by enhanced chemiluminescence (Thermo Fisher Scientific). The density of the bands was analyzed by ImageJ.

### 2.17. Statistical Analysis

Data are presented as means ± SD from three independent experiments for the *in vitro* study and in the *in vivo* study (*n* = 12). Statistical significance was calculated by Student's *t* test when comparing two sets of data. One-way ANOVA followed by the Bonferroni multiple comparison test was used for comparing more than two sets of data. *P* < 0.05 was considered statistically significant. Data were analyzed with GraphPad software 8.0 (GraphPad Software, CA, USA) and SPSS 19.0 (IBM, NY, USA).

## 3. Results

### 3.1. Identification of MSCs

**Figure 1:**
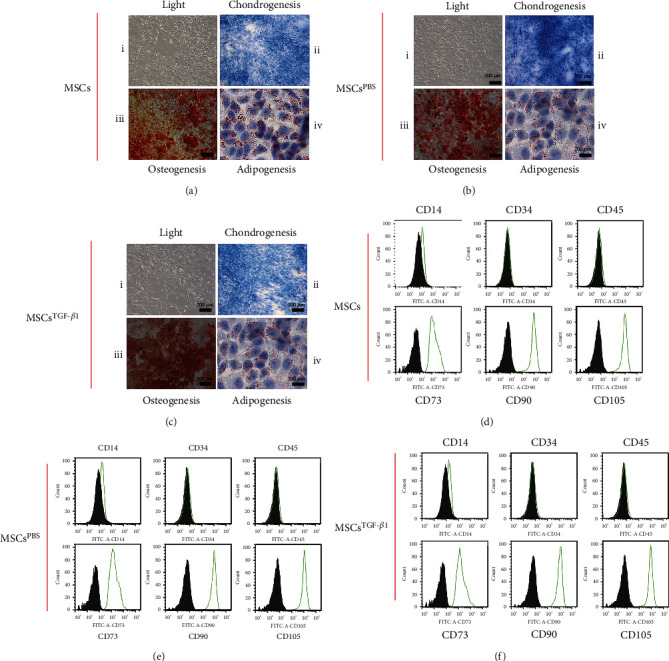
Characterization and differentiation potentials of cultured MSCs. (a) MSCs showed morphology consistent with typical MSCs (i) and could successfully differentiate into osteoblasts (ii), chondrocytes (iii), and adipocytes (iv), *n* = 3. (b, c) MSC^PBS^ and MSC^TGF-*β*1^ morphology was similar to normal MSCs (i) and could differentiate into osteoblasts (ii), chondrocytes (iii), and adipocytes (iv), *n* = 3. (d–f) Cultured MSCs were immunostained with antibodies for CD73, D90, and CD105 (positive) and CD14, CD34, and CD45 (negative). The stained cells were analyzed using flow cytometry to detect the surface markers specific to MSCs. The percentage of the cell population with positive or negative staining is represented in each figure, *n* = 3. MSCs: mesenchymal stem cells.

The cultivated normal MSCs had a morphology that was very similar to that of fibroblasts when viewed using an inverted light microscope (Figure 1(a)). These cells were all flexible and adherent, and they were long and polygonal. Furthermore, we established that MSCs are capable of differentiating into osteogenic, chondrogenic, and adipogenic lineages (Figure 1(a)). MSCs exhibited a characteristic MSC immunophenotype by expressing positive levels of CD73, CD90, and CD105 but not CD14, CD34, or CD45 (Figure 1(d)). This is known as a positive immunophenotype. After treating the MSCs with PBS and TGF-*β*1, we checked if the shape and characteristics of the cells had changed. These cells displayed a pattern characteristic of MSCs, as shown in Figures 1(b) and 1(c). They were highly positive for the markers CD73, D90, and CD105, whereas CD14, CD34, and CD45 were negative (Figures 1(e) and 1(f)). According to these data, treatment with PBS or TGF-*β*1 did not result in any changes to the typical morphology or immunotype of MSCs. In addition, it was found that MSCs treated with either PBS or TGF-*β*1 were capable of differentiating into osteogenic, chondrogenic, and adipogenic cell lines (Figures 1(b) and 1(c)). In conclusion, normal MSCs, MSCs treated with PBS, or MSCs treated with TGF-*β*1 were all plastic-adherent cells that were either long or polygonal in shape. This established a strong basis for the studies that we carried out later.

### 3.2. TGF-*β*1 Promotes SCD1 Expression and EV Release from MSCs

**Figure 2:**
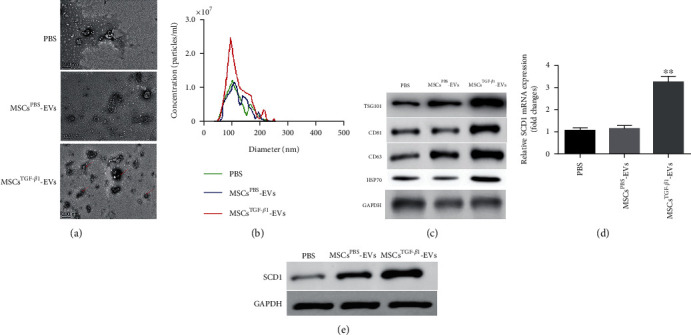
TGF-*β*1 promotes SCD1 expression and EV release from MSCs. (a) Morphology of EVs derived from MSCs treated with PBS or TGF-*β*1, *n* = 3. (b) NTA of EVs derived from the PBS, MSC^TGF-*β*1^-EV, and MSC^PBS^-EV groups, *n* = 3. (c) Western blot analysis of EV markers, including TSG101, CD81, CD63, and HSP70, *n* = 3. (d, e) The expression level of SCD1 in the MSC^TGF-*β*1^-EV and MSC^PBS^-EV groups was detected by RT-PCR (d) and western blotting (e), *n* = 3. Data are expressed as mean ± SD; ^∗∗^*P* < 0.01. MSCs: mesenchymal stem cells; EVs: extracellular vesicles.

To confirm that TGF-*β*1 could control the expression of SCD1 in MSCs, TGF-*β*1 (10 ng/mL) was used to activate MSCs. PBS-stimulated MSC-EVs (MSC^PBS^-EVs) and TGF-*β*1-stimulated MSC-EVs (MSC^TGF-*β*1^-EVs) were recovered from cell supernatants 24 h later. TEM was used to determine the morphology of the EVs, and the nanoparticle tracking analysis (NTA) method was used to quantify the EVs (Figure 2(a)). The number of MSC^TGF-*β*1^-EVs was substantially higher than MSC^PBS^-EVs (Figure 2(b)). The expression of particular EV markers such as TSG101, CD63, CD81, and HSP70 [27] in the medium was evaluated by western blot analysis and found to be higher in cells treated with TGF-*β*1 (Figure 2(c)). Compared to the MSC^PBS^-EV group, the MSC^TGF-*β*1^-EV group had higher levels of SCD1 mRNA and protein expression (Figures 2(d) and 2(e)). In conclusion, we successfully obtained MSC^TGF-*β*1^-EVs and confirmed the overexpression of SCD1 in MSC^TGF-*β*1^-EVs.

### 3.3. MSC^TGF-*β*1^-EV Transplantation Promotes Bone Fracture Repair in Mice

**Figure 3:**
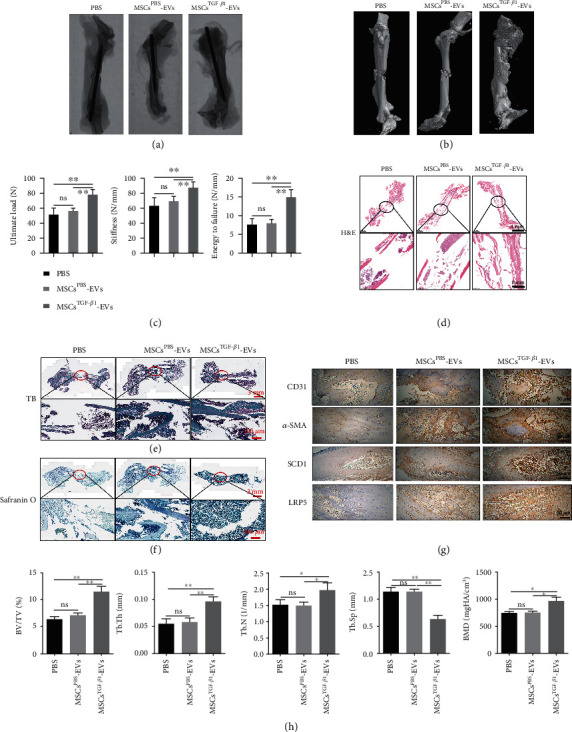
Transplanting MSC^TGF-*β*1^-EVs promotes bone fracture repair in mice. (a) Representative radiograph images of the femur fracture model in mice 21 d postfracture from the PBS, MSC^TGF-*β*1^-EV, and MSC^PBS^-EV groups, *n* = 6. (b) Representative 3D images from micro-CT scanning of the femur fracture model in mice on day 21 postfracture from the different groups, *n* = 6. (c) Detection of changes in the ultimate load, stiffness, and energy to failure, *n* = 6. (d–f) Results of H&E, TB, and Safranin O-Fast Green staining for the femur fracture model in mice on day 21 postfracture from the PBS-, MSC^TGF-*β*1^-EV-, and MSC^PBS^-EV-exposed groups, *n* = 5. (g) Immunohistochemistry of CD31, *α*-SMA, SCD1, and LRP5 for the femur fracture model in mice 21 d postfracture from the PBS-, MSC^TGF-*β*1^-EV-, and MSC^PBS^-EV-exposed groups, *n* = 6. (h) Bone volume/tissue volume (BV/TV), trabecular number (Tb.N, mm^−1^), trabecular thickness (Tb.Th, mm), trabecular spacing (Tb.Sp, mm), and bone mineral density (BMD) in the PBS-, MSC^TGF-*β*1^-EV-, and MSC^PBS^-EV-exposed groups at 21 d, *n* = 6. Data are expressed as mean ± SD; ^∗^*P* < 0.05 and ^∗∗^*P* < 0.01. ns: no significance; MSCs: mesenchymal stem cells; EVs: extracellular vesicles.

To determine whether MSC^TGF-*β*1^-EVs could influence the bone healing process *in vivo*, we created a femoral fracture model in mice and compared callus formation after injecting the mice with MSC^TGF-*β*1^-EVs, MSC^PBS^-EVs, or a PBS control. Figures 3(a) and 3(b) depict radiograph and 3D micro-CT scanned images of the femurs taken 21 d postfracture. Significant increases in the ultimate load, stiffness, and energy to failure were seen in the MSC^TGF-*β*1^-EV group compared to both the MSC^PBS^-EVs and PBS groups, indicating a considerable improvement in mechanical attributes (Figure 3(c)). Callus development and bone bridging development were evaluated using H&E, TB, and safranin O-fast green staining across the three treatment groups (Figures 3(d)–3(f)). There was a similar degree of bone repair in the PBS and MSC^PBS^-EV-treated femurs, but the femurs treated with MSC^TGF-*β*1^-EVs had more cartilaginous and osseous callus formation with bone bridging at a more advanced stage than in the control mice. Moreover, 3D micro-CT scanned images revealed that vascularization was far more advanced in the femurs treated with MSC^TGF-*β*1^-EVs than in the MSC^PBS^-EV-treated mice or the PBS control group. Bone deposition and bridging occurred more rapidly in the femurs of mice treated with MSC^TGF-*β*1^-EVs compared to mice treated with MSC^PBS^-EVs and the PBS control group. We also assessed levels of CD31, *α*-SMA, SCD1, and low-density lipoprotein receptor-related proteins (LRP) 5 at the site of injury (Figure 3(g)). The CD31 marker on endothelial cells is a reliable indicator of recent angiogenesis [28], *α*-SMA is a marker of osteoprogenitors in the periosteum during fracture healing [29], and LRP5 is used to indicate the level of osteogenesis and bone density [30]. All the markers were significantly upregulated in response to MSC^TGF-*β*1^-EV treatment at the site of the fracture. Figure 3(h) further demonstrates that compared to the MSC^PBS^-EV group and the PBS group, the BV/TV of callus in the MSC^TGF-*β*1^-EV group was considerably greater. The trabecular thickness (Tb.Th) and trabecular number (Tb.N, mm^−1^) of the fracture in the MSC^TGF-*β*1^-EV group were also significantly higher when compared to the MSC^PBS^-EV group and the PBS group. Tb.Sp in the MSC^TGF-*β*1^-EV group was lower than the MSC^PBS^-EV group and the PBS group. BMD at the fracture healing areas in the MSC^TGF-*β*1^-EV group was significantly higher than in the MSC^PBS^-EV group and the PBS group. Overall, these results indicate that transplantation with MSC^TGF-*β*1^-EVs promotes the healing of bone fractures *in vivo* more effectively than MSC^PBS^-EVs.

### 3.4. MSC^TGF-*β*1^-EVs Induce HUVEC Proliferation, Migration, and Tube Formation *In Vitro*

**Figure 4:**
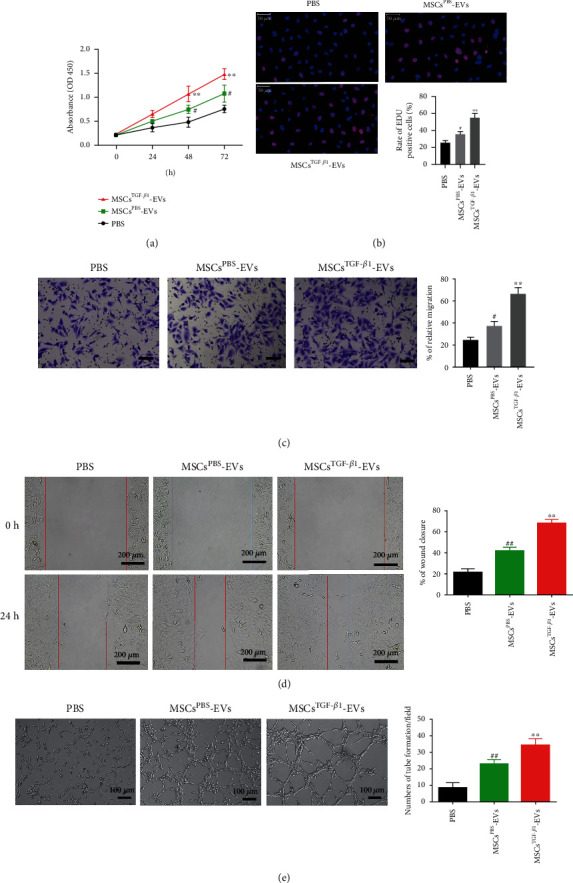
MSC^TGF-*β*1^-EVs induce HUVEC proliferation, migration, and tube formation *in vitro*. (a) Cell proliferation of HUVECs after administration of PBS, MSC^TGF-*β*1^-EVs, and MSC^PBS^-EVs as measured by CCK-8 assay, *n* = 3. (b) Cell proliferation of HUVECs measured by EdU staining, *n* = 3. (c) Representative images showing migrated HUVECs using the Transwell assay (left). Quantitative analysis of the migrated cells (right), *n* = 3. (d) Representative images showing the migration ability of HUVECs at 24 h using a wound healing assay (left). Quantitative analysis of the migration rate of HUVECs (right), *n* = 3. (e) Representative images showing tube formation in HUVECs treated with PBS, MSC^TGF-*β*1^-EVs, and MSC^PBS^-EVs (left). Quantitative analysis of the tube formation assay (right), *n* = 3. Data are expressed as mean ± SD. ^#^*P* < 0.05 and ^##^*P* < 0.01, PBS vs. MSCs^PBS^ − EVs < 0.05; ^∗∗^*P* < 0.01, MSC^PBS^-EVs vs. MSC^TGF-*β*1^-EVs. MSCs: mesenchymal stem cells; EVs: extracellular vesicles; HUVECs: human umbilical vein endothelial cells.

To better understand the role of MSC^TGF-*β*1^-EVs in fracture healing and angiogenesis, we measured proliferation, migration, and tube formation in HUVECs treated with 100 *μ*g/mL of MSC^TGF-*β*1^-EVs, MSC^PBS^-EVs, and PBS. The reaction to MSC^TGF-*β*1^-EVs was greater than the response to MSC^PBS^-EVs in terms of cell proliferation, migration, and wound closure in HUVECs compared to the PBS-treated group. After treatment with MSC^PBS^-EVs, HUVECs showed significant improvements in cell proliferation, migration, and wound closure when compared to the PBS-treated group; the response to MSC^TGF-*β*1^-EVs was larger than the response to MSC^PBS^-EVs (Figures 4(a)–4(d)). In comparison to the PBS control group, increased levels of tube formation were seen in HUVECs that had been treated with MSC^TGF-*β*1^-EVs, MSC^PBS^-EVs, or both. The highest levels of tube formation were seen in HUVECs that had been treated with MSC^TGF-*β*1^-EVs (Figure 4(e)). According to these findings, the contribution of MSC^TGF-*β*1^-EVs to fracture repair and angiogenesis is greater than that of MSC^PBS^-EVs.

### 3.5. SCD1 Is Transferred to HUVECs by EVs

**Figure 5:**
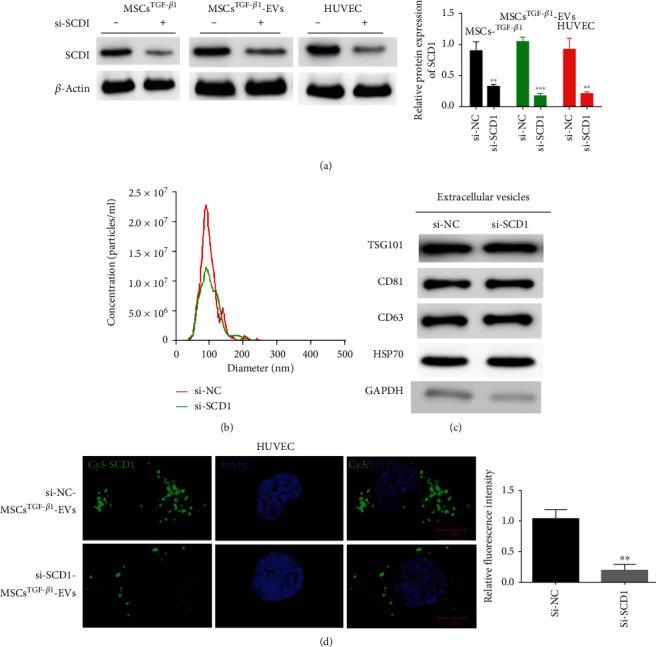
SCD1 is transferred to HUVECs by EVs. (a) The SCD1 level in MSCs^TGF-*β*1^, MSC^TGF-*β*1^-EVs transfected with si-NC and si-SCD1, target HUVECs after administrating si-NC-TGF-*β*1-EVs or si-SCD1-TGF-*β*1-EVs, and the efficiency confirmed using western blotting and RT-PCR, *n* = 3. (b) NTA of EVs derived from MSC^TGF-*β*1^-EVs transfected with si-NC and si-SCD1, *n* = 3. (c) Western blot analysis of EV proteins including TSG101, CD81, CD63, and HSP70, *n* = 3. (d) Representative images of Cy3-labeled EV-SCD1 internalized by HUVECs after administration of MSC^TGF-*β*1^-EV-si-NC or MSC^TGF-*β*1^-EV-si-SCD1 (left). Quantitative analysis of relative fluorescence intensity (right), *n* = 3. Data are expressed as mean ± SD; ^∗∗^*P* < 0.01. MSCs: mesenchymal stem cells; EVs: extracellular vesicles; HUVECs: human umbilical vein endothelial cells.

We next evaluated SCD1 for its ability to be transmitted to HUVECs via EVs. RT-PCR and western blotting confirmed that SCD1 was expressed in MSCs^TGF-*β*1^, MSC^TGF-*β*1^-EVs, and targeted HUVECs and that silencing SCD1 could lower the levels of its protein (Figure 5(a)). This demonstrates that si-NC- and si-SCD1-transfected MSC^TGF-*β*1^-EVs were delivered to the target HUVECs efficiently. Although the size of EVs produced by si-SCD1-transfected cells differed to those from si-NC-transfected cells, NTA analysis of MSC^TGF-*β*1^-EVs revealed a similar amount of EVs in both types of cells (Figure 5(b)). The expression of the EV markers TSG101, CD81, CD63, and HSP70 was similar in MSC^TGF-*β*1^-EV-si-NC and MSC^TGF-*β*1^-EV-si-SCD1 (Figure 5(b)). Cy3-labeling confirmed that the levels of EV-SCD1 in the cytoplasm of HUVECs were lower after the administration of si-SCD1 from MSC^TGF-*β*1^-EVs (Figure 5(d)). These results confirm the transfer of EV-SCD1 into HUVECs.

### 3.6. SCD1 Knockdown Reduces MSC^TGF-*β*1^-EV-Stimulated Proliferation, Angiogenesis, and Migration *In Vivo* and *In Vitro*

**Figure 6:**
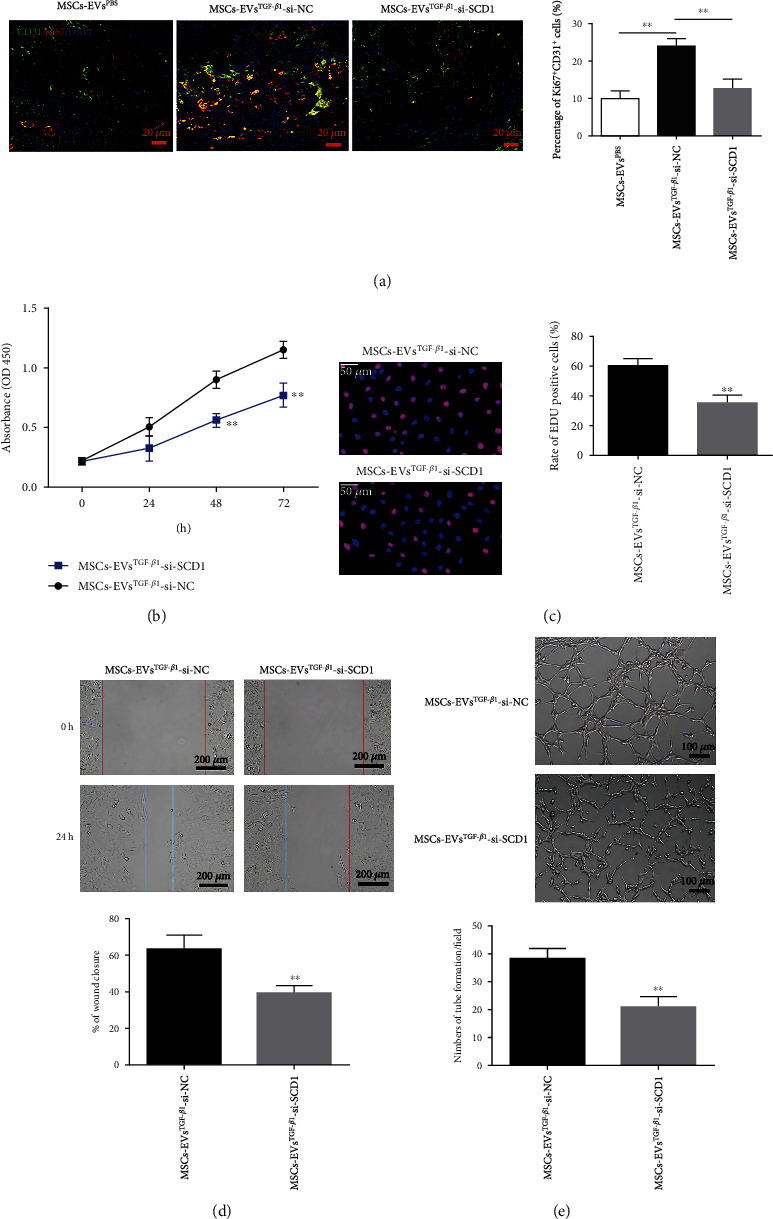
SCD1 knockdown reduces MSC^TGF-*β*1^-EV-stimulated proliferation, angiogenesis, and migration *in vivo* and *in vitro*. (a) Immunofluorescence staining of Ki-67 (red) and CD31 (green) in callus tissues from mice administered with MSC^PBS^-EVs, MSC^TGF-*β*1^-EV-si-NC, or MSC^TGF-*β*1^-EV-si-SCD1 on day 21 postfracture. Quantification of positive Ki67/CD31 cells, *n* = 3. (b–e) The functional effects of MSC^TGF-*β*1^-EV-si-SCD1 on proliferation, migration, and angiogenesis as measured by CCK-8, EdU, wound healing assays, and tube formation, *n* = 3. Data are expressed as mean ± SD; ^∗∗^*P* < 0.01. MSCs: mesenchymal stem cells; EVs: extracellular vesicles.

Whether the differential expression of SCD1 could influence the characteristics of MSC^TGF-*β*1^-EVs was then determined both *in vivo* and *in vitro*. Callus tissues from mice administered with MSC^TGF-*β*1^-EVs, MSC^TGF-*β*1^-EV-si-NC, or MSC^TGF-*β*1^-EV-si-SCD1 were probed with Ki-67 (red immunofluorescence) and CD31 (green immunofluorescence) 21 d postfracture (Figure 6(a)). The level of cell proliferation was lower in tissue treated with MSC^TGF-*β*1^-EV-si-SCD1 than in tissue treated with MSC^TGF-*β*1^-EV-si-NC. Similarly, the functional effects of silencing SCD1 *in vivo* were observed *in vitro* in HUVECs treated with either MSC^TGF-*β*1^-EV-si-NC or MSC^TGF-*β*1^-EV-si-SCD1. HUVECs treated with MSC^TGF-*β*1^-EV-si-NC showed a higher amount of cell proliferation, as evaluated by CCK-8 and EdU staining (Figures 6(b) and 6(c)). Wound healing experiments and tube formation both showed similar effects on migration (Figure 6(d)) and angiogenesis (Figure 6(e)), respectively. In conclusion, SCD1 downregulation by RNA interference reduces MSC^TGF-*β*1^-EV-mediated cell proliferation, migration, and angiogenesis *in vivo* and *in vitro*.

### 3.7. SREBP-1 Is Required for the SCD1 Transcriptional Response

**Figure 7:**
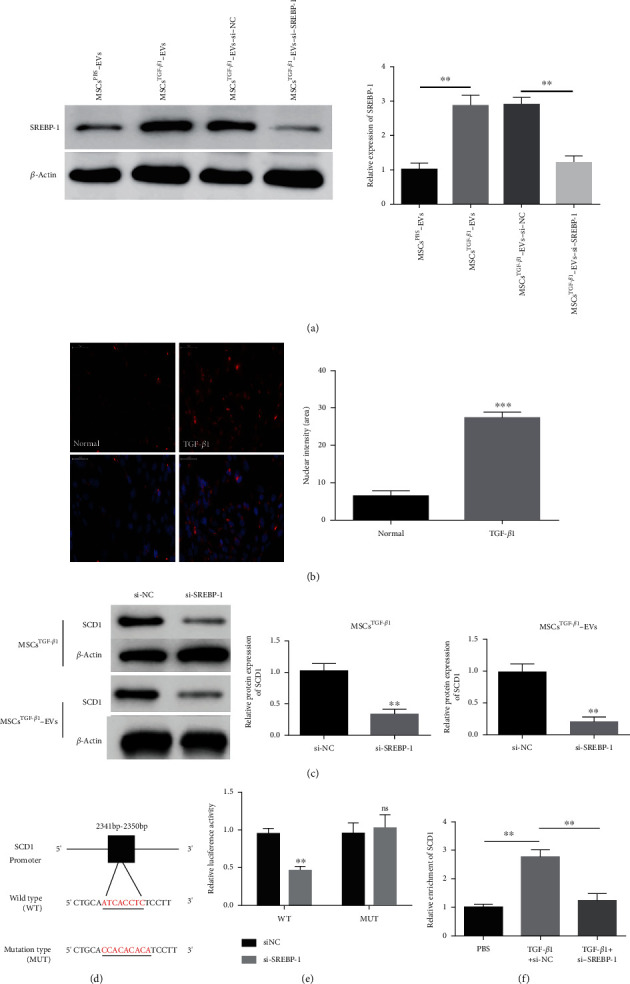
SREBP-1 is required for SCD1 transcriptional response. (a) The SREBP-1 levels in MSCs^TGF-*β*1^, MSC^TGF-*β*1^-EVs, MSC^TGF-*β*1^-EV-si-NC, or MSC^TGF-*β*1^-EV-si-SREBP-1 were assessed by western blotting, *n* = 3. (b) MSCs were treated with TGF-*β*1, and nuclear SREBP-1 translocation was assessed by immunofluorescence, with quantification shown in the bar graph, *n* = 3. (c) The SCD1 protein levels in MSCs^TGF-*β*1^ and MSC^TGF-*β*1^-EVs treated with si-NC or si-SREBP-1 were assessed by western blotting, *n* = 3. (d) Schematics of the SCD1 promoter and luciferase construct are depicted with the binding site and deletion mutation sequences. (e) Relative luciferase activity of the WT SCD1 promoter or the MUT promoter was detected, *n* = 3. (F) ChIP assay shows binding of SREBP-1 to the SCD1 promoter is enhanced by TGF-*β*1 stimulation, *n* = 3. Data are expressed as mean ± SD; ^∗∗^*P* < 0.01 and ^∗∗∗^*P* < 0.001. ns: no significance; MSCs: mesenchymal stem cells; EVs: extracellular vesicles.

We used RNA interference to silence SREBP-1 in MSC^TGF-*β*1^-EVs to determine whether SREBP-1 was involved in the effects that SCD1 had on cell proliferation and angiogenesis (Figure 7(a)). Stimulating MSCs with TGF-*β*1 causes an increase in nuclear translocation of SREBP-1 (Figure 7(b)). However, SREBP-1 silencing results in reduced expression of SCD1 in MSCs^TGF-*β*1^ and MSC^TGF-*β*1^-EVs (Figure 7(c)). The proposed SREBP-1 binding site was mutated in the promoter of SCD1, and the relative luciferase activity confirmed an interaction between SREBP-1 and SCD1 (Figures 7(d) and 7(e)). Moreover, a ChIP assay indicated that the binding of SREBP-1 to the SCD1 promoter is enhanced by TGF-*β*1 stimulation (Figure 7(f)). Overall, these results indicate that SREBP-1 regulates SCD1 expression and that this regulation is enhanced by TGF-*β*1 stimulation.

### 3.8. EV-SCD1 Promotes HUVEC Proliferation, Angiogenesis, and Migration in Association with LRP5

**Figure 8:**
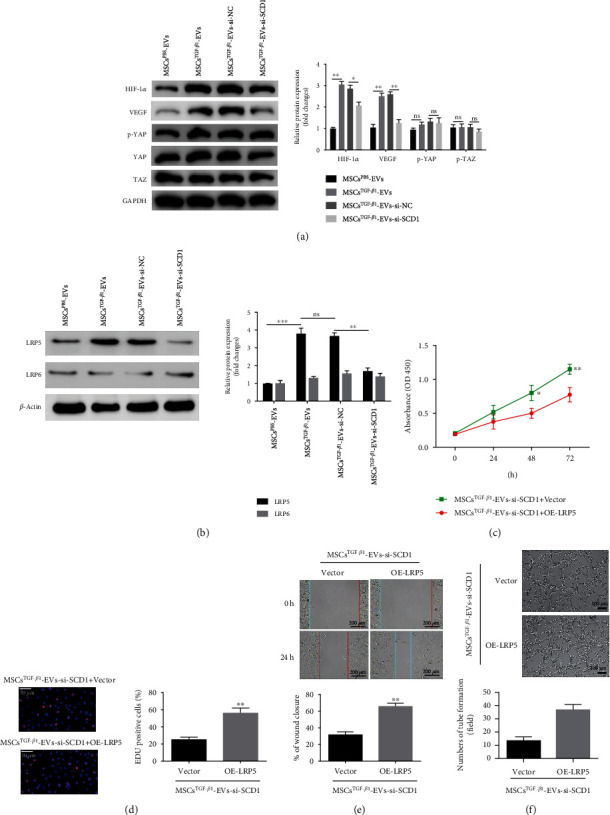
EV-SCD1 promotes HUVEC proliferation, angiogenesis, and migration by interactions involving LRP5. (a) Expression levels of HIF-1*α*, VEGF, TAZ, YAP, and p-YAP in HUVECs after transfection with MSC^PBS^-EVs, MSC^TGF-*β*1^-EVs, MSC^TGF-*β*1^-EV-si-NC, or MSC^TGF-*β*1^-EV-si-SCD1, *n* = 3. (b) Expression level of LRP5 and LRP6 in HUVECs after transfection with MSC^PBS^-EVs, MSC^TGF-*β*1^-EVs, MSC^TGF-*β*1^-EV-si-NC, or MSC^TGF-*β*1^-EV-si-SCD1, *n* = 3. (c–f) CCK-8 (c), EdU (d), scratch wound assays (e), and tube formation (f) were used to verify the functional role of LRP5 on cell proliferation, migration, and angiogenesis in HUVECs, *n* = 3. Data are expressed as mean ± SD; ^∗^*P* < 0.05, ^∗∗^*P* < 0.01, and ^∗∗∗^*P* < 0.001. ns: no significance; MSCs: mesenchymal stem cells; EVs: extracellular vesicles; HUVECs: human umbilical vein endothelial cells.

During fracture healing, the activation of the Hippo signaling system [31] and the HIF-1/VEGF38 pathway [32] has been shown to increase angiogenesis and vascular remodeling. We first examined changes in key proteins in angiogenesis-related pathways after MSC^TGF-*β*1^-EVs, MSC^TGF-*β*1^-EV-si-NC, and MSC^TGF-*β*1^-EV-si-SCD1 were administered to HUVECs. The levels of HIF-1*α* and VEGF in HUVECs in the MSC^TGF-*β*1^-EV group were significantly increased. In contrast, the expression of SCD1 was suppressed, and the protein expression of HIF-1*α*/VEGF was lowered. However, the phosphorylation and protein levels of YAP and TAZ did not change significantly (Figure 8(a)). This suggests that the HIF-1*α*/VEGF pathway is necessary for EV-SCD1-induced HUVEC angiogenesis. Next, the expression levels of specific proteins involved in fracture healing were measured after MSC^TGF-*β*1^-EVs, MSC^TGF-*β*1^-EV-si-NC, or MSC^TGF-*β*1^-EV-si-SCD1were administered to HUVECs. The protein expression level of LRP5 in HUVECs was significantly increased by the addition of MSC^TGF-*β*1^-EVs and MSC^TGF-*β*1^-EV-si-NC whereas MSC^TGF-*β*1^-EV-si-SCD1 had little impact (Figure 8(b)), and there was no major change in the protein expression of LRP6. To further investigate the association between SCD1 and LRP5 expressions, the effect of overexpressing LRP5 on the proliferation (Figures 8(c) and 8(d)), migration (Figure 8(e)), and angiogenesis (Figure 8(f)) of HUVECs was measured by CCK-8, EdU, wound healing assays, and tube formation. The overexpression of LRP5 resulted in higher levels of cell proliferation, migration, and angiogenesis in HUVECs (Figures 8(c)–8(f)). Based on these findings, it appears that LRP5 is involved in the process by which MSC^TGF-*β*1^-EV-SCD1 promotes proliferation, angiogenesis, and migration in HUVECs.

## 4. Discussion

Regenerative medicine involving the use of MSCs offers great potential in optimizing the healing of bone fractures and bone abnormalities that are difficult to cure [33, 34]. However, there are restrictions on the use of MSCs in clinical applications because they are difficult to transplant, have a short life span, and have the potential to induce cancer [35]. MSC-EVs provide a useful alternative that resolves the pluripotent issues associated with stem cells [36]. Because of their ability to impact various biological processes, including both angiogenesis and osteogenesis, MSCs have been selected to release EVs to aid the repair process during bone healing [14, 37, 38]. This paracrine effect has been exploited in several studies to deliver specific repair factors to the site of bone injury [39, 40].

In this study, we examined the role of MSC^TGF-*β*1^-EVs in angiogenesis and fracture repair. TGF-*β*1 is a pleiotropic cytokine that is known to modulate MSCs by regulating differentiation and homeostasis and has been used successfully to stimulate the repair of fractures [41, 42]. In agreement with the published studies, we found that MSC^TGF-*β*1^-EVs enhanced callus development in an *in vivo* bone fracture model. When injected locally at the site of injury, MSC^TGF-*β*1^-EVs were able to promote bone healing, which manifested as increased bone volume density, trabecular number, trabecular thickness, and BMD, while reducing trabecular spacing in a mouse fracture model. In addition, the results of the immunohistochemical study showed an increased expression of CD31, *α*-SMA, SCD1, and LRP5 at the site of injury, which indicated that levels of angiogenesis and osteogenesis were increased in response to the paracrine effect of MSC^TGF-*β*1^-EVs.

LRP5 and LRP6 form a complex with Wnt and Frizzled to control the activation of *β*-catenin phosphorylation [43]. The Wnt/*β*-catenin pathway is a conserved cascade of signaling pathways involved in the proliferation, differentiation, and regulation of stem cells [44]. A recent study has found that SCD1 provides a feedback loop to control the level of Wnt/*β*-catenin signaling by modulating LRP5 and LRP6 expressions [45]. In this study, we found that LRP5 and SCD1 protein expressions were significantly upregulated after MSC^TGF-*β*1^-EV treatment at the site of injury. The addition of MSC^TGF-*β*1^-EVs or MSC^TGF-*β*1^-EV-si-NC greatly raised the expression level of LRP5 in HUVECs, whereas the addition of MSC^TGF-*β*1^-EV-si-SCD1 had minimal impact on the level of expression. The protein expression of LRP6 in HUVECs was relatively unaffected, indicating that the expression of SCD1 upregulates LRP5. LRP5 seemed to have a greater impact on cell proliferation, migration, and angiogenesis. Several studies have also observed differences in the signaling activity of LRP5 and LRP6 [46, 47]. Wnt3a is known to regulate bone metabolism in conjunction with LRP5 and LRP6 [45]. LRP6 is believed to have a more active role in the Wnt3a-mediated differentiation of osteoblasts. However, dramatic losses in bone density are observed when LRP5 is mutated [48], which signifies that LRP5 and LRP6 play different roles in osteogenesis [49].

In our study, we found that SREBP-1 interacts with SCD1 to stimulate cell proliferation and angiogenesis. Furthermore, we discovered that knocking down SREBP-1 significantly reduced SCD1 expression in MSCs^TGF-*β*1^ and MSC^TGF-*β*1^-EVs, thereby abolishing the effects of TGF-*β*1-EVs. This observation supports the results found in previous studies where SREBP-1 interacts with SCD1 to control Wnt signaling and LRP5 and LRP6 expressions [45]. SREBP-1 is thought to be activated by TGF-*β*1 [19]; therefore, our results indicate that MSC^TGF-*β*1^-EVs promote bone healing through the activation of SREBP-1-mediated SCD1 transcription and the subsequent upregulation of LRP5.

There are some limitations to our research, such as the absence of validated clinical data. In addition, the results of our studies have indicated that suppressing SCD1 leads to a decrease in the amount of proliferation, angiogenesis, and migration of HUVECs that is mediated by MSC^TGF-*β*1^-EVs in both *in vivo* and *in vitro* conditions; however, we have not investigated the effects of SCD1 overexpression. We also did not analyze the regulatory effects of SREBP-1 on LRP5. Furthermore, there is no comprehensive investigation of the particular mechanisms performed by SREBP-1 and LRP5 in animal models, which may be the primary focus of research in the near future. Studies in a follow-up phase could potentially include investigations into connected pathways.

## 5. Conclusions

**Figure 9:**
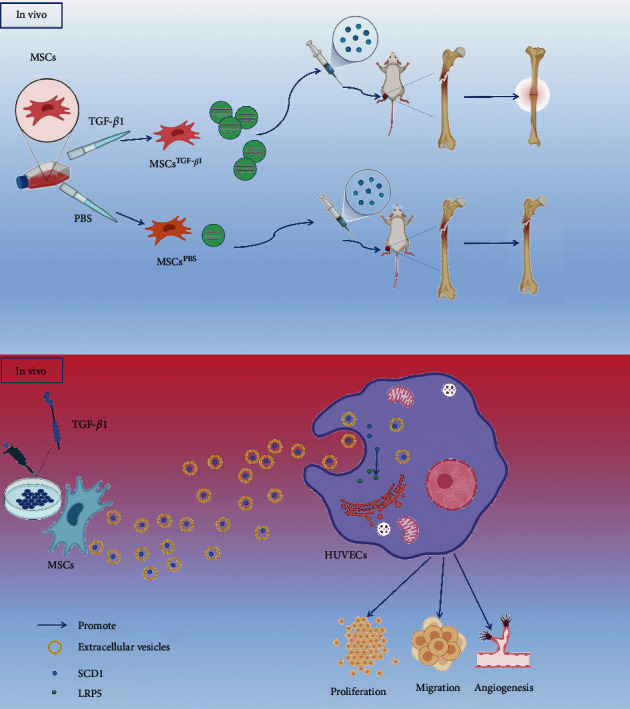
Schematic representation depicts that bone fracture healing is promoted in mice after transplantation with MSC^TGF-*β*1^-EVs *in vivo.* MSC^TGF-*β*1^-EV-SCD1 promotes HUVEC proliferation, angiogenesis, and migration by interactions involving LRP5 *in vitro*. MSCs: mesenchymal stem cells; EVs: extracellular vesicles; HUVECs: human umbilical vein endothelial cells.

In summary, the paracrine processes of MSC^TGF-*β*1^-EVs in a mouse model of bone fracture and HUVECs were examined in our study. The primary objective of this study was to compare the effects of MSC^TGF-*β*1^-EVs and MSC^PBS^-EVs on the healing of bone fractures. We found that MSC^TGF-*β*1^-EVs were more effective at promoting angiogenesis, proliferation, and migration than MSC^PBS^-EVs. In addition, the findings of this study reveal a strategy through which MSC^TGF-*β*1^-EVs enhance bone fracture repair via SCD1 in a chain reaction of contacts including SREBP-1 and LRP5. Our findings lead us to the conclusion that preconditioning with TGF-*β*1 is an efficient strategy for the therapeutic optimization of MSC-EVs in bone fracture healing (Figure 9).
